# Intimate partner violence and associated factors among reproductive age women in Liberia: a cross-sectional study using a recent Liberian demographic and health survey

**DOI:** 10.1186/s12905-022-01830-x

**Published:** 2022-06-17

**Authors:** Menen Tsegaw, Bezawit Mulat, Kegnie Shitu

**Affiliations:** 1 Department of Health Education and Behavioral Sciences, Institute of Public Health, College of Medicine and Health Sciences, University of Gondar, Gondar, Ethiopia; 2 Department of Human Physiology, School of Medicine, College of Medicine and Health Sciences, University of Gondar, Gondar, Ethiopia; 3 Department of Public Health, College of Medicine and Health Sciences, Ambo University, Ambo, Ethiopia

**Keywords:** Intimate partner violence, Liberia, Ever-married women

## Abstract

**Background:**

Intimate partner violence (IPV) is a major public health problem and a violation of women's human rights. Almost one third of women aged 15–49 years who have been in a relationship have experienced to some form of physical and/or sexual violence by their intimate partner worldwide.

**Objective:**

The study aimed to assess the prevalence of intimate partner violence within the last 12 months and associated factors among reproductive aged women in Liberia.

**Method:**

This study was based on a large community-based cross-sectional survey, Liberia Demographic Health Survey (LDHS), conducted From October 16, 2019, to February 12, 2020, in Liberia. The 2019–20 LDHS used a stratified two-stage cluster design. Multivariable logistic regression was used to identify independent intimate partner violence among reproductive age women in Liberia and to control confounders. Adjusted odds ratio and confidence interval (CI) were used to declare statistical significance in the final model. Those variables with *p* value < 0.05 were considered as statistically significant.

**Result:**

The overall prevalence of IPV within the last 12 months was 44.74% (42.73–46.77). age of the women 41% (AOR = 0.59, 95%CI 0.37–0.93), 42% (AOR = 0.58, 95%CI 0.35–0.94), and 59% (AOR = 0.41, 95%CI 0.25–0.68) among women with in the age group of 35–39, 40–44 and 45–49 respectively, south central region (AOR = 0.71, 95%CI 0.52–0.96), women’s primary education (AOR = 1.28, 95%CI 1.01–1.63), female household head (AOR = 0.77, 95%CI 0.61–0.97), husbands higher education (AOR = 0.62, 95%CI 0.39–0.99), positive wife beating attitude (AOR = 1.57, 95%CI 1.29–1.90), husband drinks (AOR = 2.59, 95%CI 2.14–3.15) and Women’s decision making autonomy (AOR = 0.75, 95%CI 0.61–0.93) were significantly associated with IPV.

**Conclusion:**

The prevalence of IPV in Liberia was high. Socio-demographic characteristics of women, husbands education, sex of household head, having a positive attitude towards wife-beating, partner’s alcohol drinking habit and women empowerment was significantly associated with IPV in Liberia. Policymakers and program designers have to take into account those factors when they design interventions to reduce IPV in Liberia.

## Introduction

According to World Health Organization (WHO), Intimate Partner Violence (IPV) i**s** an intentional act of an intimate partner or ex-partner that causes physical, sexual, or psychological harm, including physical aggression, sexual coercion, psychological abuse, and controlling behaviors [1, 2]. IPV is a major public health problem and a violation of women's human rights, almost one third (27%) of women aged 15–49 years who have been in a relationship have experienced some form of physical and/or sexual violence by their intimate partner worldwide [3]. Intimate partner violence is a public health issue in all over the world [4]. Gender-based violence cases have surged during the Corona virus Disease (COVID-19) pandemic, increasing women's risk of acquiring Human Immunodeficiency Virus/Acquired Immune Deficiency Syndrome (HIV/AIDS), and reducing women’s access to gender-based violence as well as HIV and other sexual and reproductive health services [5–7]. IPV has mental and physical health consequences on women, particularly it leads to depressive symptoms, loss of social and professional networks by causing to feel stigmatization and absent from work as well as they may prefer to be alone [8–10]. Intimate partner violence is a public health problem in sub-Saharan Africa [11, 12]. Many studies done on intimate partner violence had revealed that older age, higher educational status of the women, urban residence, having educated husband, being exposed to media (radio, TV or reading newspaper/magazine) were significantly associated with decreased IPV whereas, alcohol abuse by the partner, women’s positive attitude towards wife beating and polygynous marriage were factors significantly associated with increased likelihood of being exposed to intimate partner violence [13–17]. The life time prevalence of intimate partner violence among ever married women in Liberia has increased from 49% in 2013 to 60% in 2019/20 [18]. In addition to IPV, Sexual violence and gender-based violence (SGBV) against women has been a predominant problem in Liberia during the era of Liberia’s civil war as well as during the COVID-19 pandemic [19]. Liberia has established a revised Gender Policy and National Action Plan with the great emphasis on addressing gender based violence among women [20–22]. However, there were challenges to the implementation and enforcement of gender and human rights-related laws in which gender inequality and women’s marginalization take the lion share for hindrance of policy implementation in addition to the multi-sectoral nature of those policies that made its implementation complex [20, 21]. The factors that are significantly associated with intimate partner violence among married reproductive age women in Liberia were not known. Therefore, this study aimed to assess the prevalence of intimate partner violence within the last 12 months and associated factors among ever-married reproductive-age women in Liberia using the Liberia Demographic Health Survey 2019/20.

## Methods

### Study design and setting

This study was based on a large community-based cross-sectional survey, Liberia Demographic Health Survey (LDHS), conducted From October 16, 2019, to February 12, 2020, in Liberia. The 2019–2020 LDHS used a stratified two-stage cluster design, the first stage involved clusters, and the second stage involved systematic sampling of households. Liberia is West African country bounded by Sierra Leone to the Northwest, Guinea to the North, Côte d’Ivoire to the east, and the Atlantic Ocean to the South and west. The total population of Liberia was 5.18 million in 2021. The 2019–2020 Liberia DHS used a standardized module of questions designed to obtain information on the extent to which women in Liberia experience domestic violence. These questions asked women about their experience of both intimate partner violence by perpetrators other than husbands and other intimate partners. The questionnaire was administered on 50% subsample of households selected for the men’s survey. Only one eligible woman aged 15–49 per household was randomly selected for the survey. In total, 3120 women aged 15–49 years completed the module with a response rate of 98.5%. From a total of 3120 reproductive age women 15–49 years invited for the domestic violence module, we have included 2331 married reproductive age women and after we weighted the sample the final sample size becomes 2100. All ever-married reproductive aged women who had reported their experience of IPV were included in the study. However, those reproductive aged women who had never married/single and who did not report their IPV experience were excluded from the study.

### Data analysis

Stata version 14.0 was used for statistical analysis. Descriptive studies like frequency count and proportion for categorical data were used to summarize descriptive data. Bivariable logistic regression was used to select candidate variables for multivariable logistic regression. In the Bivariable logistic regression, those variables having a *p* value of less than 0.2 were considered as candidate variables for multivariable logistic regression analysis. Multivariable logistic regression was used to identify independent predictors of intimate partner violence among reproductive-age women in Liberia and to control confounders. We have conducted multi-variable logistic regression by entering all variables at a time and taking those variables having *p* value of less than 0.05 as statistically significant. Adjusted odds ratio and confidence interval (CI) were used to declare statistical significance in the final model. Multi-colinearity was assesses and there was no any potential multicollinearity in which the VIF was 1.1.

### Variables of the study

#### Dependent variable

*Intimate partner violence* experience of ever-married women of one or more of spousal emotional, physical, or sexual violence within the last 12 months [11, 16, 18].. In the 2019–2029 LDHS, women were asked questions beginning with ‘Does/did your husband/partner ever…’ Women who answered yes to any specific question were asked about frequency of the action in the last 12 months (often/sometimes/not in that time). The three types of spousal violence were combined into a single spousal violence variable with binary outcomes whether a woman had ever experienced least one type of spousal violence (“yes/no) within the last 12 months. A woman was considered as experienced IPV within the last 12 months if she said yes to at least one form of violence 12 months prior to the survey. Series of questions were asked for each woman on physical, sexual and emotional violence as follows;

*Physical spousal violence* to identify physical spousal violence, women were asked to confirm that whether their husband push, shake, or throw something, slap, twist arm or pull hair, punch with his fist or with something that could hurt, kick, drag, beat up, try to choke, burn, threaten attack with a knife, gun, or any other weapon within the last 12 months [11, 16, 18].

*Sexual spousal violence* to identify sexual spousal violence, women were asked to confirm that whether their husband physically force to have sexual intercourse with him even when they did not want to, physically force them to perform any other sexual acts they did not want to, or force them with threats or in any other way to perform sexual acts they did not want to [11, 16, 18].

*Emotional spousal violence* to identify emotional spousal violence, women were asked to confirm that whether their husband say or do something to humiliate in front of others, threaten to hurt or harm them or someone close to them, or insult or make feel bad about themselves [11, 16, 18].

#### Independent variables

Independent variables of the study were extracted from the LDHS 2019/2020 data. Socio-demographic characteristics of the mother (Age of the women, religion, region, educational level, occupation, residence, wealth index), sex of household head(headed by female, headed by male), husbands education, husbands working status, attitude towards wife-beating (positive, Negative), cigarette smoking (yes, no), drinking alcohol (yes, no), currently pregnant, women decision making autonomy ( yes, no) and media exposure was computed as a composite variable which includes frequency of reading newspaper/magazine, frequency of listening to radio, frequency of watching TV, if a women was exposed to at least one form of media with the last week then she will say “yes” otherwise “no” were included in the study. Wealth index were categorized as poorest, poorer, middle, richer and richest wealth quintiles as per the DHS standard of Liberia. Religion was initially categorized as Christian, Muslim, traditional religion no religion traditional (0.47), and no religion (0.83) was less than 5% we recode religion into Christian and Muslim by grouping traditional and no religion under Muslim religion y giving the name as Muslim/other. Those variables were identified as factors associated with IPV after reviewing different literature [13, 15, 16, 23–26].

## Results

### Socio-demographic characteristics of reproductive age women in Liberia

The mean age of women was 33 years with a standard deviation of 8.67 and age range of 15–49 years. Out of 2100 participants included in the study, 42.97% of women reside in south-central region of Liberia, 43.31% of women have no formal education, 71.42% were working, 84.78% were Christians, 53.54% were from urban and 23.28% were from poorest households (Table 1).

**Table 1:** Socio demographic characteristics of reproductive age women and their association with intimate partner violence within the last 12 months in Liberia using LDHS 2019/20 (n = 2100)

Variables	Intimate partner violence		Total	Percent	*p* Value
	Yes	No			
*Age of the mother*					
15–19	62	32	94	4.5	< 0.001
20–24	175	134	309	14.7	
25–29	190	205	395	18.8	
30–34	185	205	390	18.5	
35–39	164	245	409	19.5	
40–44	107	150	257	12.3	
45–49	83	163	246	11.7	
*Region*					
North western	96	121	217	10.3	0.113
South central	428	474	902	43.0	
South eastern A	66	82	148	7.1	
South eastern B	62	57	119	5.6	
North central	314	400	714	34.0	
*Women educational level*					
No education	388	521	909	43.3	0.001
Primary	246	234	480	22.9	
Secondary	299	327	626	29.8	
Higher	33	52	85	4.00	
*Working status*					
Yes	685	815	1500	71.4	0.427
No	282	318	600	28.6	
*Religion*					
Muslim/other	137	182	319	15.2	0.330
Christian	829	952	1781	84.8	
*Residence*					
Urban	548	576	1124	53.5	0.366
Rural	418	558	976	46.5	
*Wealth index*					
Poorest	218	271	489	23.3	0.896
Poorer	195	235	430	20.5	
Middle	200	233	433	20.6	
Richer	201	181	382	18.2	
Richest	152	214	366	17.4	
*Media exposure*					
No	347	404	751	35.8	0.625
Yes	619	730	1349	64.2	
*Currently pregnant*					
Yes	109	79	188	9.0	0.006
No	857	1055	1912	91.0	
*Sex of household head*					
Male	729	759	1488	70.9	0.001
Female	237	375	612	29.1	
*Women decision making autonomy (n* = *1780)*					
No	281	250	531	29.8	< 0.001
Yes	561	688	1249	70.2	
*Husbands education (n* = *1780)*					
No education	211	240	451	25.4	0.054
Primary	114	117	230	12.9	
Secondary	388	392	780	43.8	
Higher	86	124	210	11.8	
Don’t know	44	64	108	6.1	
*Husband current working*					
No	82	89	171	8.1	0.003
Yes	884	1045	1929	91.9	
*Attitude towards wife beating*					
Negative	508	753	1261	60.1	< 0.001
Positive	458	380	838	39.9	
*Husband drinks alcohol*					
No	466	798	1264	60.2	< 0.001
Yes	500	336	836	39.8	
*Cigarette smoking*					
No	949	1129	2078	99.0	0.354
Yes	17	5	22	1.0	

### Prevalence of intimate partner violence among reproductive age women

The overall prevalence of intimate partner violence among women in the last 12 months was 44.74% (42.73–46.77). The 12 months prevalence of physical, emotional and sexual violence was 34.15% (32.25–36.10), 34.49 (32.59–36.45) and 6.56 (92.35–94.37) respectively. The most common form of violence that women experienced in the last 12 month were emotional violence followed by physical violence (Table 2).

**Table 2:** Shows the percentage of intimate partner violence within the last 12 months among reproductive age women in Liberia (n = 2100)

variables	Weighted frequency	Weighted percent (95% CI)
*Physical violence*		
Yes	725	34.15 (32.25–36.10)
No	1375	65.85 (63.89–67.75)
*Emotional violence*		
Yes	732	34.49 (32.59–36.45)
No	1368	65.51 (63.55–67.41)
*Sexual violence*		
Yes	146	6.56 (92.35–94.37)
No	1954	93.44 (5.62–7.64)
*IPV within the last 12 months*		
Yes	966	44.74 (42.73–46.77)
No	1134	55.26 (53.23–57.26)

### Results of bivariable analysis

Bivariable logistic regression was fitted to identify candidate variables for multivariable logistic regression. Age of the women, educational level, region, currently pregnant, sex of household head, husbands/partner’s education, husband drinks alcohol, women’s autonomy for decision making, and attitude towards wife-beating, were included in Bivariable regression analysis.

### Predictors of intimate partner violence in Liberia

A multivariable logistic regression model was fitted to identify independent predictors of intimate partner violence in Liberia. In multivariable logistic regression age of the women, region, sex of household head, husbands/partners education, and women decision making autonomy, and were negatively and significantly associated with intimate partner violence in the last 12 months. However, positive attitude towards wife beating, husband drinks alcohol and women’s education were positively and significantly associated with intimate partner violence among married reproductive-aged women in Liberia.

The odds of intimate partner violence in the last 12 months were lowered by 41% (AOR = 0.59, 95%CI 0.37–0.93), 42% (AOR = 0.58, 95%CI 0.35–0.94), and 59% (AOR = 0.41, 95%CI 0.25–0.68) among women with in the age group of 35–39, 40–44 and 45–49 years respectively as compared with those found within the age group of 15–19 years.

The odds of intimate partner violence was lowered by 29% (AOR = 0.71, 95%CI 0.52–0.96) among women who reside in south central region as compared with north westerns.

The odds of intimate partner violence was lowered by 23% (AOR = 0.77, 95%CI 0.61–0.97) among households headed by female as compared with those headed by males.

The odds of intimate partner violence was lowered by 38% (AOR = 0.62, 95%CI 0.39–0.99) among women whose husband has a higher educational level as compared with those whose husband has no education.

The odds of intimate partner violence was 1.57 (AOR = 1.57, 95%CI 1.29–1.90) times higher among women having a positive attitudes towards wife-beating as compared with those having a negative attitudes.

The odds of intimate partner violence was 2.59 (AOR = 2.59, 95%CI 2.14–3.15) times higher among women whose husband/partner drinks alcohol as compared with their counterparts.

Women’s decision-making autonomy decreases the odds of intimate partner violence by 25% (AOR = 0.75, 95%CI 0.61–0.93) as compared with those who have no decision-making power.

The odds of intimate partner violence was 1.28 (AOR = 1.28, 95%CI 1.01–1.63) times higher among women with primary education as compared to those with no formal education (Table 3).

**Table 3:** Factors associated with intimate partner violence within the last 12 months among reproductive-age women in Liberia (n = 2100)

Variables	COR (95%CI)	*p* Value	AOR (95%CI)	*p* Value
*Age of the women*				
15–19	1		1	
20–24	0.94 (0.62–1.44)	0.793	1.08 (0.68–1.74)	0.730
25–29	0.70 (0.46–1.06)	0.092	0.75 (0.47–1.19)	0.236
30–34	0.69 (0.45–1.05)	0.083	0.77 (0.49–1.23)	0.277
35–39	0.49 (0.32–0.74)	0.001	0.59 (0.37–0.93)*	0.026
40–44	0.48 (0.31–0.74)	0.001	0.58 (0.35–0.94)*	0.029
45–49	0.32 (0.21–0.49)	< 0.001	0.41 (0.25–0.68)*	0.001
*Region*				
North western	1		1	
South central	0.90 (0.69–1.17)	0.439	0.71 (0.52–0.96)*	0.028
South eastern A	0.98 (0.75–1.31)	0.937	0.77 (0.56–1.08)	0.130
South eastern B	1.27 (0.96–1.68)	0.094	0.92 (0.66–1.27)	0.607
North central	0.96 (0.74–1.24)	0.734	0.80(0.59–1.09)	0.157
*Religion*				
Muslim/other	1			
Christian	1.12 (0.89–1.41)	0.330	–	
*Residence*				
Urban	1			
Rural	0.92 (0.78–1.09)	0.336	–	
*Wealth index*				
Poorest	1			
poorer	1.01 (0.81–1.24)	0.895	–	
Middle	0.98 (0.78–1.23)	0.874	–	
Richer	1.06 (0.80–1.39)	0.689	–	
Richest	0.87 (0.63–1.21)	0.408	–	
*Women educational level*				
No education	1		1	
Primary	1.46 (1.20–1.78)	< 0.001	1.28 (1.01–1.63)*	0.040
Secondary	1.29 (1.05–1.59)	0.015	1.22 (0.93–1.61)	0.148
Higher	1.03 (0.59–1.81)	0.921	1.34 (0.64–2.81)	0.432
*Respondent currently working*				
No	1			
Yes	0.93 (0.77–1.12)	0.427	–	
*Currently pregnant*				
No			1	
Yes	1.46 (1.11–1.90)	0.006	1.08 (0.80–1.47)	0.598
*Sex of household head*				
Male	1		1	
Female	0.77 (0.62–0.88)	0.001	0.77 (0.61–0.97)*	0.026
*Husbands education*				
No education	1		1	
Primary	1.13 (0.86–1.49)	0.372	0.90 (0.67–1.22)	0.506
Secondary	1.14 (0.91–1.42)	0.245	0.95 (0.74–1.22)	0.703
Higher	0.68 (0.47–0.99)	0.048	0.62 (0.39–0.99)*	0.043
Don’t know	0.90 (0.62–1.33)	0.605	0.85 (0.56–1.28)	0.434
*Husband current working*				
No	1			
Yes	0.99 (0.69–1.41)	0.959		
*Media exposure*				
No	1			
Yes	1.04 (0.88–1.23)	0.625	–	
*Attitude towards wife beating*				
Negative	1		1	
Positive	1.73 (1.46–2.04)	< 0.001	1.57 (1.29–1.90)*	< 0.001
*Husband drinks alcohol*				
No	1		1	
Yes	2.48 (2.09–2.94)	< 0.001	2.59 (2.14–3.15)*	< 0.001
*Smokes cigarettes*				
No	1			
Yes	1.51 (0.63–3.67)	0.358	–	
*Women decision making autonomy*				
No	1		1	
Yes	0.66 (0.55–0.81)	< 0.001	0.75 (0.61–0.93)*	0.008

*p* < 0.001 was labeled for *p* = 0.000, (–): Not applicable for AOR, (*): statistically significant at *p* < 0.05.

## Discussions

This study aimed to assess the prevalence of intimate partner violence in the last 12 months and associated factors among women in Liberia. The overall prevalence of intimate partner violence among women in the last 12 months was 44.74 (42.73, 46.77). This finding is higher than the studies done in Namibia, Sub-Saharan Africa, India and Ethiopia [11, 27–29]. The possible reason for the discrepancy might be difference in sample size, study population, socio-cultural differences among countries and differences in the tools those studies used to measure IPV. The study conducted in Namibia included pregnant women attending ANC and used a small sample size (n = 386) as compared with our study which included all ever-married women (married, divorced, widowed or separated) and large sample size which is a nationally representative sample. The study conducted in Namibia used validated questionnaire that was previously applied in an antenatal clinic study in South Africa which is slightly different from the standardized tool that we have used to assess IPV. The study conducted in India measured IPV by using information obtained from ever-married women who reported violence by their husbands and by others unlike the present study which included violence report by their intimate spouse/husband. The study conducted in sub-Saharan Africa included 16 countries in which there may be socio-cultural differences within and among countries affecting the prevalence of IPV.

The prevalence of physical, emotional, and sexual violence in the last 12 months were 34.15 (32.25, 36.10), 34.49 (32.59, 36.45 and 6.56 (92.35, 94.37) respectively. The finding is lower than the study conducted in Saudi-Arabia [30]. However, this finding is higher than the study conducted in India [28]. The possible reason for the discrepancy might be sample size difference (n = 403 in Saudi-Arabia), the instrument used, and the type of study design. The current study used a large community based cross-sectional study unlike the study done in Saudi-Arabia used institution-based cross-sectional design which may affect women’s disclosure of abuse and they have used small sample size as compared with our study (n = 403). Emotional violence was the most common form of violence among ever-married women in Liberia followed by physical violence. This finding is in line with the studies conducted in Lagos, Saudi-Arabia and Ethiopia [23, 30, 31].

Age of the women was significantly associated with intimate partner violence. Older women were less likely to experience intimate partner violence as compared with younger women. This finding is inconsistent with the studies conducted in Ethiopia, Namibia, and Bangladeshi [23, 27, 32]. The possible reason for the discrepancy might be the difference in study population used, in which those studies were conducted on pregnant women which might increase IPV. Pregnancy might increase the likelihood of experiencing IPV [33, 34]. The other possible reason might be older women are mature and can discuss openly with their spouses. Older women are emotionally stable as compared with those of younger ages which are characterized as fire age group, for example if a certain dispute occurs in the house older women will tolerate and discuss the issues with their husbands. Older aged women might be more experienced in solving conflict than younger ones; therefore older women may solve certain arguments before they lead to violence by discussing issues critically with their husband.

Region of the women were significantly associated with intimate partner violence. Women residing in south central were less likely to experience IPV as compared with those live in north western region of Liberia. This might be due to the fact that IPV has spatial variation within the country and across countries [35]. Regions within the country might have different socio-demographic makeup that might affect the prevalence of IPV. South central region was also the one in which relatively largest number of women married educated husband were living which might have its own part in resulting lower risk of being exposed to IPV in the region as compared with other regions. Relatively large numbers of reproductive aged women above the age group of 15–19 years were also residing in south central as compared with the other region of Liberia which might contribute to the low level of occurrence of IPV in south central region than other regions.

Sex of household head was significantly associated with IPV. Households headed by female were less likely to experience IPV as compared with those headed by male. The possible reason might be that when a woman heads a household she will have the power to make a decision on all important issues and she will be empowered which in turn reduces her likelihood of experiencing IPV. If a woman is the head of the household, she will have autonomous control and ownership of all assets in the household including media platforms in which exposure to media can help the women to develop a negative attitude towards wife-beating and will have justifications for rejecting wife-beating [36].

Women's education was significantly associated with IPV. Women with education were more likely to face IPV as compared with those with no education. This finding is inconsistent with the studies conducted in Ethiopia and Sub-Saharan Africa [14, 35]. The possible reason might be, even though education is important to change an attitude and behavior we cannot conclude that knowing is enough to get the actual intended behavior. There are factors that influence a person’s behavior in addition to education like socio-cultural factors especially gender-based violence is highly influenced and significantly varied across cultures and societal norms [37].

Husbands’ education was significantly associated with IPV. Women whose husbands have higher educational levels were less likely to experience IPV as compared to those with no education. This is consistent with the studies done in Ghana, and Rwanda [15, 16]. Education is a powerful instrument to shape attitudes and change behavior. Educated individuals can get information from different sources about domestic violence and other health-related information in general [23]. The other possible reason might be that educated men may give freedom to their wife as compared with uneducated ones and education may also help to change men attitude towards gender roles and norms as well as the traditional perception towards gender equality [31]].

The women´s attitude towards wife-beating was significantly associated with IPV. Having a positive attitude towards wife-beating increases women's experience of IPV. This finding is consistent with the study done in Zimbabwe and Ethiopia [24, 38]. The possible reason might be women having positive attitude towards wife beating may simply accept beating as just part of the normal life of couples and may become victims of IPV.

Husband’s alcohol drinking habit was significantly associated with IPV. A woman whose husband drinks alcohol was more likely to experience IPV than their counterparts. This is consistent with the studies done in Ethiopia, Ghana, Rwanda, Nigeria, and Uganda [15, 16, 26, 39, 40]. The possible reason might be that alcohol can affect both physical and psychological functioning and it may also disturb the marital relationship in general by inviting them to engage in conflict rather than resolving it. Alcohol might also reduce judgmental capability and rational interpretation as well as an understanding of stimulus around the person. Another possible explanation might be related with financial issue within the family, drinking habit of the man may result in financial catastrophe of the family which may in turn causes nagging and argument between couples. In addition to their drinking habit alcohol drinkers may face alcohol related problems like cohabitation of unmarried partners which may perpetuate the occurrence of conflict between the couples and IPV at large.

Women decision making autonomy was significantly associated with IPV. Women who were empowered to make decision in all matters were less likely to experience IPV as compared with women who have no decision making autonomy. This finding is in line with the studies done in Zimbabwe, Sub-Saharan Africa, and India [14, 28, 38]. The possible reason might be women who have a decision making power are empowered to decide on important issues and may protect their right. Empowered women might more likely use media which may increase her awareness on social and gender issues and may change her attitude [14]. Empowered women have a greater self-esteem control over their own lives and their surrounding environment,as a result she will have a low probability of being exposed to IPV [41].

### Strength

The study used nationally weighted representative data that better reflects the proportion of married women experiencing intimate partner violence and its associated factors at the national level and regional level. The other strength of the current study is the use of a large sample size that can help to increase the statistical power and validity of the study. Utilization of large sample size and nationally representativeness of DHS data helps to generalize to the population of Liberia.

## Limitations

Due to the cross-sectional nature of the study it’s impossible to establish a temporal relationship between intimate partner violence and its predictors. Moreover, since this study was solely dependent on the secondary data set, some important variables like socio-cultural factors that would affect intimate partner violence may not be found.

## Conclusion

The prevalence of IPV within the last 12 months was high in Liberia. Age of the women, region, women’s education, husbands education, sex of household head, having positive attitude towards wife beating, partners alcohol drinking habit and women decision making autonomy were significantly associated with IPV in Liberia. Policymakers and program designers have to take into account those factors when they design public health interventions to reduce IPV in Liberia. Gender violence prevention programmers must prioritize approaches that include sexual education and empowerment of women. Different gender based organizations should work in collaboration with men to reduce IPV and promote gender equitable relationships between couples.

## Data Availability

The datasets used and/or analyzed during the current study are publicly available and can access it from https://dhsprogram.com/data/available-datasets.cfm.

## Abbreviations

AOR: Adjusted odds ratio
CI: Confidence interval
COR: Crude odds ratio
LDHS: Liberia Demographic Health Survey
